# Thermal Inactivation of Hepatitis E Virus: A Narrative Review

**DOI:** 10.3390/v17050702

**Published:** 2025-05-14

**Authors:** Tatsuo Kanda, Hiroaki Okamoto

**Affiliations:** 1 Division of Gastroenterology and Hepatology, Uonuma Institute of Community Medicine, Niigata University Medical and Dental Hospital, Uonuma Kikan Hospital, Minami-Uonuma 949-7302, Japan; 2 Division of Virology, Department of Infection and Immunity, Jichi Medical University School of Medicine, 3311-1 Yakushiji, Shimotsuke-shi 329-0498, Tochigi, Japan; hokamoto@jichi.ac.jp

**Keywords:** blood products, cell culture, heat stability, heat inactivation, hepatitis E virus, infectivity, pig liver, swine bioassay

## Abstract

Hepatitis E virus (HEV) infection is an emerging infectious disease. HEV-1 and HEV-2 infect humans through contaminated water and foods, mainly in developing countries. HEV-3 and HEV-4 also infect humans through contaminated food and are thought to be zoonotic infections occurring in both developing and developed countries. A vaccine for hepatitis E is licensed in only limited countries. The inactivation of infectious HEV is very important to ensure the safety of drinking water and foods. HEV-3 and HEV-4 RNA have been detected in some pig liver products, and it is possible that these foods may represent an infectious source of HEV. In this article, previous publications on the heat inactivation and heat stability of HEV are collected, and we discuss the present assessment of the heat inactivation of HEV. The thermal stability of HEV infection in cell culture systems and pig bioassays has been demonstrated, while the efficacy of the method of thermal inactivation using plasma products has not yet been established. Here, we propose that the treatment of HEV-contaminated foods at 95 °C for 10 min is one of the safest options for the inactivation of HEV.

## 1. Introduction

Hepatitis E virus (HEV) infection causes acute hepatitis, including fulminant hepatitis, and chronically infects immunocompromised humans [1,2,3,4,5,6,7]. HEV infection also carries extrahepatic manifestations, such as Guillain–Barré and Miller Fisher syndromes [8]. The broad-spectrum antiviral ribavirin, available with or without interferon, is effective in eradicating HEV in chronic hepatitis E. However, it cannot be used to treat some populations due to its adverse events; for example, pregnant women and patients with anemia [9,10,11]. Other antivirals against HEV are in development. It is important to prevent HEV infection [11].

HEV is a positive-sense, single-stranded RNA virus with an approximate genome length of 7.2 kb [11]. The HEV particle consists of two forms: the quasi-enveloped form (eHEV: the membrane-associated form) and the nonenveloped form (neHEV: the non-membrane-associated form). eHEV is coated within the lipid membrane, and, like the exosome, it is released from hepatocytes into the blood stream and the cell-culture-conditioned medium. neHEV is formed from eHEV by the detergents of bile acids and is present in the bile and feces. In general, HEV replicates in hepatocytes. Human HEV has four major genotypes, as follows: HEV-1 and HEV-2 infect humans through contaminated water and foods, mainly in developing countries; HEV-3 and HEV-4 also infect humans through contaminated foods, are thought to be zoonotic infections, and are found in both developing and developed countries [11,12,13].

At present, HEV’s transmission routes, environmental stability, heterogeneity, and treatment are still not fully understood [14]. The World Health Organization (WHO) estimates that approximately 20 million HEV infections occur every year, resulting in about 3.3 million symptomatic cases worldwide [15]. The WHO reported that hepatitis E caused approximately 44,000 deaths in 2015 [15]. Thus, HEV infection is one of the most significant emerging infectious diseases in the developing and developed worlds.

The inactivation of infectious HEV is very important to ensure the safety of drinking water and foods. It has been reported that several methods, such as dry heating, liquid heating, high-pressure processing, solvent treatment, detergent treatment, chlorine and ultraviolet treatment, and virus-removal filtering (nanofilters) have been developed for the inactivation and/or removal of HEV from different sources, such as clotting factor concentrates [16,17], plasma products [18], blood products [19,20,21], pig livers [22,23,24], drinking water [25,26], and cell cultures [27,28,29]. However, the results of these studies are varied. Further investigations into HEV inactivation and removal are urgently needed in the near future.

A vaccine for hepatitis E is not yet available in any country other than China and Pakistan [30]. Nucleic acid testing (NAT) is useful for the prevention of the transfusion transmission of HEV infection [31,32,33,34,35,36,37]. In Japan, universal individual donation NAT screening, with its urgent requirement for blood donations, was commenced in 2020 [36,37]. HEV infects humans through contaminated water and foods. The consumption of uncooked or undercooked pig liver or intestines seems to be a major source of HEV-3/HEV-4 infection in Japan [38,39,40], Germany [41,42], Italy [43], Colombia [44], the United States [22,45,46], the Netherlands [47], India [48], France [49], and Ireland [50] (Table 1). To prevent HEV infection, water and food should be provided after the inactivation of HEV. In this review, previous publications on the heat inactivation and heat stability of HEV have been collected, and we discuss the present assessment of the heat inactivation of HEV.

## 2. Thermal Stability of Hepatitis E Virus in Cell Culture Systems

Huang et al. [51] reported that four HEV-1 or HEV-4 strains—G93-1, G93-3, and G93-4 or G93-2, respectively, which were originally isolated in the human lung carcinoma A549 cell line from the feces of four patients with acute hepatitis E at Guangzhou Municipal Infectious Diseases Hospital in 1993—were not resistant to heat applied at 56 °C for 30 min. Their heat stability was determined by a microculture titration method using A549 cells. The authors evaluated the cytopathic effect (CPE) via virus titration, and their titers were all <1.0 (50% tissue culture infective dose/0.025 mL) [51]. They used an L9(34)-positive-cross design, after serial 10-fold dilution was performed in a growth medium, to select the best propagation conditions [51].

Emerson et al. [52] examined the thermal stability of the HEV-1 Akluj and Sar55 strains, which were isolated from patients with hepatitis in India and Pakistan, respectively. Further, HEV-2 Mex 14 stock in HepG2/C3A cells was assessed via residual infectivity, which was determined by immunofluorescent microscopy. Almost all of the Akluj strain was inactivated after treatment at 56 °C for 1 h, and the 50% inactivation temperature was between 45 °C and 50 °C; however, approximately 1% of the Akluj strain was still infectious, even after heating at 56 °C for 1 h [52].

Almost 50% of Sar55 was inactivated by incubation at 56 °C for 1 h, and 96% of Sar55 was inactivated by incubation at 60 °C for 1 h. Mex 14 was not inactivated at 56 °C for 1 h, but about 80% of Mex 14 was inactivated at 60 °C for 1 h [52]. For reference, 60 °C is 140 °F, which is close to the internal temperature of rare steak (135 °F) [52]. Immunofluorescence microscopy showed 300–400 and 500–600 positive HepG2/C3A cells infected with the HEV-1 Akluj and Sar55 strains, respectively, after treatment at 4 °C for 1 h; ~50 and 2000–2500 positive HepG2/C3A cells infected with HEV-1 Akluj and Sar55 strains, respectively, after treatment at 50 °C for 1 h; and ~20 or ~40 positive HepG2/C3A cells infected with HEV-2 Mex 14 after treatment at 4 °C or 50 °C for 1 h, respectively [52].

Tanaka et al. [53] examined the thermal stability of the HEV-3b (JE03-1760F) strain, which was originally isolated from fecal specimens of a 67-year-old Japanese patient with acute hepatitis E and chronic renal failure, in PLC/PRF/5 cells. They measured HEV RNA levels in the culture medium. HEV RNA was measured via real-time detection RT-PCR.

They were diluted to 6.0 × 10^4^ copies per well and inoculated on PLC/PRF/5 cells [53]. When the same amount of HEV inoculum was incubated at 95 °C for 10 min, 95 °C for 1 min, or 70 °C for 10 min prior to inoculation on PLC/PRF/5 cells, HEV RNA was not detectable throughout the observation period of 50 days following inoculation. Following the incubations at 56 °C for 30 min and room temperature (25 °C) for 30 min, HEV RNA was first detected on day 20 and day 16, respectively [53].

Yunoki et al. [18] examined the thermal stability of pig HEV strains (HEV-3e, HEV-3a, HEV-3b, and HEV-4c) in A549 cells [18]. In their study, they used HEV-3e, HEV-3a, HEV-3b, and HEV-4c, respectively, showed 7.5 or 7.7, 7.2 or 8.4, 6.3 and 7.0, 7.0/7.4, 7.4, 6.8 or 7.2 log copies per mL of the viral titer HEV genome and 4.8 or 5.8, 4.8 or 5.3, 3.8 and not available (N/A), 4.8, 3.2, 3.8, or 3.8 log dilution non-detectable end-points per mL of HEV infectivity [18]. They reported that residual infectivity was not detected with a log reduction factor (LRF) >4.0 after treatment at 80 °C for 24 h in any of the samples. HEV infectivity was detected in all the samples that were treated at 60 °C for 72 h [18].

PCR-based methods may not be suitable for use in differentiating between infectious and non-infectious viruses [54]. Schielke et al. [54] applied an RNase A treatment followed by quantitative real-time RT-PCR in order to distinguish disassembled from intact viral particles. They examined the thermal stability of wild boar liver containing HEV-3i isolate wbGER27 in the three cell lines PLC/PRF/5, A549, and HepG2, followed by viral antigen detection by immunofluorescence. An analysis by quantitative real-time RT-PCR revealed 5 × 10^8^ genome equivalents (GEs) per mL in the liver suspension. Treatment with RNase A prior to RNA extraction resulted in 3 × 10^7^ GE/mL corresponding to intact viral particles in the liver suspension. They assessed HEV stability at different temperatures for 1 min and found that the incubation at 80 °C and 85 °C for 1 min resulted in 2.47-log_10_ and 2.58-log_10_ reductions, respectively, whereas the incubations at 90 °C and 95 °C for 1 min resulted in 3.58-log_10_ and 3.67-log_10_ reductions, respectively [54].

Johne et al. [28] examined the thermal stability of the HEV-3 strain 47832c in A549/D3, using the titration method and via the counting of focus-forming units (ffu) by immunofluorescence, leading to measurements of HEV infectivity. HEV infectivity lasts up to 21 days at 37 °C, up to 28 days at room temperature, and up to at least 56 days at 4 °C. After 1 min of heating at 80 °C, 85 °C, and 90 °C, no ffus were observed [28]. In their experiments, they used HEV, which had a viral infectivity of at least 3.7 log ffus in infection experiments.

Imagawa et al. [55] examined the thermal stabilities of HEV-3 strain 83-2 and HEV-4 strain 121-12, which were isolated from pig liver, in PLC/PRF/5 cells. The concentrations of virus culture supernatants used were 1.74 × 10^8^ copies per mL for HEV-3 and 1.83 × 10^8^ copies per mL for HEV-4. They observed the differences in the thermal stability of HEV-3 and HEV-4. Both HEV-3 and HEV-4 were inactivated in culture supernatants heated at >65 °C for 5 min and >80 °C for 1 min and in minced meat treated at 70 °C for 5 min. They also examined the internal temperature of pork during cooking. Boiling showed superior heating efficacy to roasting [55].

Stunnenberg et al. [56] examined the thermal stability of HEV-3c strain 14-16753 and HEV-3e strain 14-22707 in a PLC/PRF/5 cell culture medium and in extracts from inoculated pork products after the thermal food-processing step. Either HEV-3c or HEV-3e with an unknown titer was used, for which the number of viral particles has not yet been estimated. They performed an immunofluorescence detection assay using A549/D3 cells and a real-time RT-PCR assay. For the liver homogenate, the greatest degree of the inactivation of HEV-3c and HEV-3e was observed following treatment at 71 °C for 5 min or longer [56].

**Figure 1:**
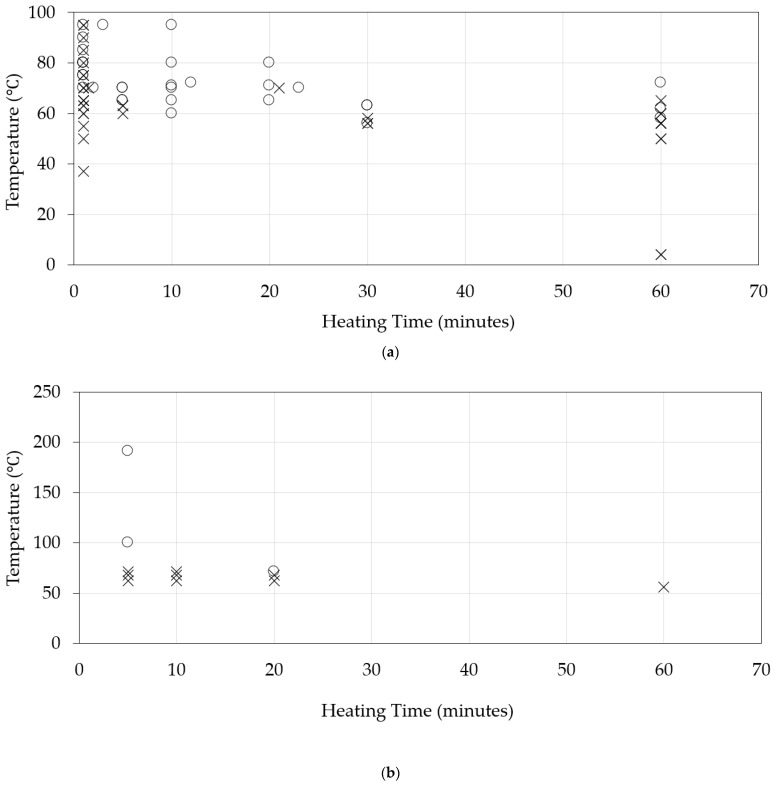
The thermal inactivation of the hepatitis E virus (HEV). (**a**) Results from cell culture systems. Refs. [18,28,50,51,52,53,54,55,56,57,58,60]. (**b**) Results from pig bioassays. Refs. [22,23]. The representative data, equal to or less than 60 min, were used. Circle, inactivation; cross, non-inactivation.

Monini et al. [57] examined the thermal stability of HEV-3c strain IT-13 and HEV-3e strain IT-12 in A549 cells via real-time RT-PCR detection focusing on HEV RNA in the cell supernatant. Five hundred μL aliquots of the viral stocks of HEV-3e or HEV-3c containing 2.54 × 10^5^ or 1.02 × 10^6^ genome copies/mL, respectively, were conserved in 0.5 mL tubes. HEV RNA was not reduced by treatment at −20 °C for 12 weeks. Heat treatment at 56 °C for 12 min did not influence the in vitro infectivity of HEV-3c, but heat treatment at 56 °C for 6 min or 12 min reduced HEV-3e infectivity. The heat inactivation of HEV at 93 °C for 1 min and 3 min left no residual HEV RNA [57]. A summary of the thermal stability of HEV in cell culture systems is shown in Table 2. Examinations for the thermal stability of HEV in cell culture systems are useful for the study of the thermal stability of HEV (Figure 1a).

**Table 2:** Representative reports of the thermal stability of the hepatitis E virus (HEV) in cell culture systems.

HEV Genotypes	Cell Lines	Detection Methods	Treatment Temperature and Duration	Completely Inactivation	Refs.
HEV-1/4	A549	TCID_50_/0.025 mL, CPE	56 °C for 30 min	Yes	[51]
HEV-1	HepG2/C3A	Focus-forming units (ffu)	4 °C for 1 h	No	[52]
HEV-1	HepG2/C3A	Focus-forming units (ffu)	50 °C for 1 h	No	[52]
HEV-1	HepG2/C3A	Focus-forming units (ffu)	56 °C for 1 h	No	[52]
HEV-1	HepG2/C3A	Focus-forming units (ffu)	60 °C for 1 h	No	[52]
HEV-2	HepG2/C3A	Focus-forming units (ffu)	4 °C for 1 h	No	[52]
HEV-2	HepG2/C3A	Focus-forming units (ffu)	50 °C for 1 h	No	[52]
HEV-2	HepG2/C3A	Focus-forming units (ffu)	56 °C for 1 h	No	[52]
HEV-2	HepG2/C3A	Focus-forming units (ffu)	60 °C for 1 h	No	[52]
HEV-3b	PLC/PRF/5	Real-time RT-PCR for HEV RNA in CM	56 °C for 30 min	No	[53]
HEV-3b	PLC/PRF/5	Real-time RT-PCR for HEV RNA in CM	70 °C for 10 min	Yes	[53]
HEV-3b	PLC/PRF/5	Real-time RT-PCR for HEV RNA in CM	95 °C for 1 min	Yes	[53]
HEV-3b	PLC/PRF/5	Real-time RT-PCR for HEV RNA in CM	95 °C for 10 min	Yes	[53]
HEV-3	A549	Real-time RT-PCR for cellular HEV RNA	80 °C for 24 h	Yes	[18]
HEV-3	A549	Real-time RT-PCR for cellular HEV RNA	60 °C for 72 h	No	[18]
HEV-3i	PLC/PRF/5, A549, HepG2	Real-time RT-PCR, Focus-forming units (ffu)	95 °C for 1 min	No	[54]
HEV-3i	PLC/PRF/5, A549, HepG2	Real-time RT-PCR, Focus-forming units (ffu)	90 °C for 1 min	No	[54]
HEV-3i	PLC/PRF/5, A549, HepG2	Real-time RT-PCR, Focus-forming units (ffu)	85 °C for 1 min	No	[54]
HEV-3i	PLC/PRF/5, A549, HepG2	Real-time RT-PCR, Focus-forming units (ffu)	80 °C for 1 min	No	[54]
HEV-3i	PLC/PRF/5, A549, HepG2	Real-time RT-PCR, Focus-forming units (ffu)	75 °C for 1 min	No	[54]
HEV-3i	PLC/PRF/5, A549, HepG2	Real-time RT-PCR, Focus-forming units (ffu)	70 °C for 1 min	No	[54]
HEV-3i	PLC/PRF/5, A549, HepG2	Real-time RT-PCR, Focus-forming units (ffu)	No heat	No	[54]
HEV-3c	A549/D3	Focus-forming units (ffu)	37 °C for 21 days	No	[28]
HEV-3c	A549/D3	Focus-forming units (ffu)	Room temperature for 28 days	No	[28]
HEV-3c	A549/D3	Focus-forming units (ffu)	4 °C for 56 days	No	[28]
HEV-3c	A549/D3	Focus-forming units (ffu)	No heat for 1 min	No	[28]
HEV-3c	A549/D3	Focus-forming units (ffu)	37 °C for 1 min	No	[28]
HEV-3c	A549/D3	Focus-forming units (ffu)	50 °C for 1 min	No	[28]
HEV-3c	A549/D3	Focus-forming units (ffu)	55 °C for 1 min	No	[28]
HEV-3c	A549/D3	Focus-forming units (ffu)	60 °C for 1 min	No	[28]
HEV-3c	A549/D3	Focus-forming units (ffu)	65 °C for 1 min	No	[28]
HEV-3c	A549/D3	Focus-forming units (ffu)	70 °C for 1 min	No	[28]
HEV-3c	A549/D3	Focus-forming units (ffu)	75 °C for 1 min	No	[28]
HEV-3c	A549/D3	Focus-forming units (ffu)	80 °C for 1 min	Yes	[28]
HEV-3c	A549/D3	Focus-forming units (ffu)	85 °C for 1 min	Yes	[28]
HEV-3c	A549/D3	Focus-forming units (ffu)	90 °C for 1 min	Yes	[28]
HEV-3c	A549/D3	Focus-forming units (ffu)	70 °C for 1.5 min	No	[28]
HEV-3c	A549/D3	Focus-forming units (ffu)	70 °C for 2 min and longer	Yes	[28]
HEV-3k	PLC/PRF/5	Real-time RT-PCR for HEV RNA in CM	56 °C for 1 h	No	[55]
HEV-3k	PLC/PRF/5	Real-time RT-PCR for HEV RNA in CM	58 °C for 30 min	No	[55]
HEV-3k	PLC/PRF/5	Real-time RT-PCR for HEV RNA in CM	58 °C for 1 h	Yes	[55]
HEV-3k	PLC/PRF/5	Real-time RT-PCR for HEV RNA in CM	60 °C for 5 min	No	[55]
HEV-3k	PLC/PRF/5	Real-time RT-PCR for HEV RNA in CM	60 °C for 10 min and longer	Yes	[55]
HEV-3k	PLC/PRF/5	Real-time RT-PCR for HEV RNA in CM	62 °C for 60 min	Yes	[55]
HEV-3k	PLC/PRF/5	Real-time RT-PCR for HEV RNA in CM	63 °C for 1 min	No	[55]
HEV-3k	PLC/PRF/5	Real-time RT-PCR for HEV RNA in CM	63 °C for 5 min	No	[55]
HEV-3k	PLC/PRF/5	Real-time RT-PCR for HEV RNA in CM	63 °C for 30 min	Yes	[55]
HEV-3k	PLC/PRF/5	Real-time RT-PCR for HEV RNA in CM	65 °C for 1 min	No	[55]
HEV-3k	PLC/PRF/5	Real-time RT-PCR for HEV RNA in CM	65 °C for 5 min	Yes	[55]
HEV-3k	PLC/PRF/5	Real-time RT-PCR for HEV RNA in CM	70 °C for 1 min	Yes	[55]
HEV-3k	PLC/PRF/5	Real-time RT-PCR for HEV RNA in CM	70 °C for 5 min	Yes	[55]
HEV-3k	PLC/PRF/5	Real-time RT-PCR for HEV RNA in CM	75 °C for 1 min	Yes	[55]
HEV-3k	PLC/PRF/5	Real-time RT-PCR for HEV RNA in CM	80 °C for 1 min	Yes	[55]
HEV-4	PLC/PRF/5	Real-time RT-PCR for HEV RNA in CM	60 °C for 1 min	No	[55]
HEV-4	PLC/PRF/5	Real-time RT-PCR for HEV RNA in CM	63 °C for 1 min	No	[55]
HEV-4	PLC/PRF/5	Real-time RT-PCR for HEV RNA in CM	63 °C for 5 min	No	[55]
HEV-4	PLC/PRF/5	Real-time RT-PCR for HEV RNA in CM	63 °C for 30 min	Yes	[55]
HEV-4	PLC/PRF/5	Real-time RT-PCR for HEV RNA in CM	65 °C for 1 min	No	[55]
HEV-4	PLC/PRF/5	Real-time RT-PCR for HEV RNA in CM	65 °C for 5 min	Yes	[55]
HEV-4	PLC/PRF/5	Real-time RT-PCR for HEV RNA in CM	70 °C for 1 min	No	[55]
HEV-4	PLC/PRF/5	Real-time RT-PCR for HEV RNA in CM	70 °C for 5 min	Yes	[55]
HEV-4	PLC/PRF/5	Real-time RT-PCR for HEV RNA in CM	75 °C for 1 min	Yes	[55]
HEV-4	PLC/PRF/5	Real-time RT-PCR for HEV RNA in CM	80 °C for 1 min	Yes	[55]
HEV-3c/3e	A549/D3	Focus-forming units (ffu)	4 °C for 1 week	No	[56]
HEV-3c/3e	A549/D3	Focus-forming units (ffu)	10 °C for 1 week	No	[56]
HEV-3c/3e	A549/D3	Focus-forming units (ffu)	21 °C for 1 week	No	[56]
HEV-3c/3e	A549/D3	Focus-forming units (ffu)	21 °C for 2 weeks	Yes	[56]
HEV-3c/3e	A549/D3	Focus-forming units (ffu)	65 °C for 10 min	Yes	[56]
HEV-3c/3e	A549/D3	Focus-forming units (ffu)	65 °C for 20 min	Yes	[56]
HEV-3c/3e	A549/D3	Focus-forming units (ffu)	71 °C for 10 min	Yes	[56]
HEV-3c/3e	A549/D3	Focus-forming units (ffu)	71 °C for 20 min	Yes	[56]
HEV-3e	A549/D3	Focus-forming units (ffu)	80 °C for 10 min	Yes	[56]
HEV-3e	A549/D3	Focus-forming units (ffu)	80 °C for 20 min	Yes	[56]
HEV-3c/3e	A549	Real-time RT-PCR for HEV RNA in CM	4 °C for 12 weeks	No	[57]
HEV-3c/3e	A549	Real-time RT-PCR for HEV RNA in CM	-20 °C for 12 weeks	No	[57]
HEV-3c/3e	A549	Real-time RT-PCR for HEV RNA in CM	56 °C for 1 h	No	[57]
HEV-3c/3e	A549	Real-time RT-PCR for HEV RNA in CM	65 °C for 1 h	No	[57]
HEV-3c/3e	A549	Real-time RT-PCR for HEV RNA in CM	72 °C for 12 min	Yes	[57]
HEV-3c/3e	A549	Real-time RT-PCR for HEV RNA in CM	72 °C for 1 h	Yes	[57]
HEV-3e	A549	Real-time RT-PCR for HEV RNA in CM	95 °C for 1 min	No	[57]
HEV-3c/3e	A549	Real-time RT-PCR for HEV RNA in CM	95 °C for 3 min	Yes	[57]
HEV-3c	A549	Focus-forming units (ffu)	(Sausage) 70 °C for 21 min in water bath	No	[58]
HEV-3c	A549	Focus-forming units (ffu)	(Sausage Core) 70 °C for 23 min in water bath	Yes	[58]

Refs., references; TCID_50_, 50% tissue culture infective dose; CPE, cytopathic effect; CM, conditioned medium. HEV-3 subtypes were classified, according to the reference [59].

## 3. Thermal Stability of Hepatitis E Virus in Pig Bioassays

Feagins et al. [45] reported that pigs inoculated with two of the three HEV RNA-PCR-positive pig-liver homogenates became HEV-infected, as evidenced by the detection of fecal virus shedding, viremia, and seroconversion. This model is a useful pig bioassay that can evaluate infectious HEV. As positive controls, pigs were each inoculated i.v. with 1 mL of standard pig HEV-3 infectious stock with an infectious titer of 5 × 10^4.5^ 50% pig infectious doses [45].

Feagins et al. [22] examined the thermal stability of commercial pig liver homogenates (FL58 and FL91) containing HEV-3 using a pig bioassay. All the pigs were inoculated intravenously with 2 mL of liver homogenates from HEV-negative or HEV-contaminated livers. Four out of five of the pigs inoculated with HEV-3-positive liver homogenates incubated at 56 °C for 1 h became infected, meaning that incubation at 56 °C for 1 h cannot inactivate HEV-3, although stir-frying the meat at 191 °C (an internal temperature of 71 °C) for 5 min (n = 5) or boiling in water for 5 min (n = 5) led to no infection [22]. The starting/spiking concentrations of HEV RNA were not indicated [22].

Barnaud et al. [23] examined the industrial processing of pork products that had been experimentally contaminated with HEV-3e at various times and temperatures, and after treatment, the presence of residual infectious HEV particles was measured by real-time RT-PCR and in vivo experimental pig bioassays. The level of the HEV contamination of the liver was estimated to be 10^8^ copies of HEV GE/g [23]. They revealed that heating food to an internal temperature of at least 71 °C for 20 min is necessary to completely inactivate HEV, but HEV fecal excretion and HEV seroconversion were observed in the foods after heating to an internal temperature of 71 °C for 10 min. All the pigs were inoculated intravenously in the ear with 2 mL of virus suspension [23].

Pig bioassays are useful for the study of the thermal stability of HEV (Figure 1b); however, this approach may not be particularly convenient due to its costs and certain ethical problems.

## 4. Thermal Stability of Hepatitis E Virus in Plasma Products

Yunoki et al. [18] reported dry heating at 80 °C to be effective for the inactivation of HEV, supporting the previous report that freeze-drying followed by dry heat treatment at 80 °C for 72 h was effective in inactivating a wide range of enveloped and nonenveloped viruses [61]. The inactivation patterns of HEV at 60 °C with albumin and fibrinogen were similar to those of canine parvovirus, which is used as a model of heat-resistant viruses, suggesting that HEV is a heat-resistant virus [18]. Yunoki et al. [18] used 4–6 non-detectable, end-point infectious HEV (log/mL). It is insufficient to inactive HEV in plasma products/plasma derivatives featuring factor (F)VIII by heating at 60 °C alone [62,63]. Yunoki et al. [62] initially used a 6–8 log virus infectivity of HEV-3. Satake et al. reported that the lowest HEV RNA dose associated with transfusion-transmitted hepatitis E presence in fresh frozen plasma is 36,000 IU of HEV RNA [64]. As the pasteurization of an HEV-positive plasma derivative at 60 °C for 10 h leads to an effective reduction in infectivity, resulting in a von Willebrand Factor (VWF)/FVIII product with a margin of safety suitable only for HEV [64,65,66,67], more safety methods should be developed to inactivate HEV. Dähnert et al. [65] performed pig bioassays, and pasteurization at 60 °C for 10 h of HEV-positive plasma derivatives could lead to the effective reduction of infectivity. The HEV copy numbers in the liver homogenate had an HEV load of 3.9 × 10^3^ RNA copies/μL, and the intermediate spike of 1:11 had an approximately 10-fold lower HEV RNA concentration [65].

## 5. Discussion

The thermal stability of HEV in cell cultures seems to depend on HEV strains/(sub)genotypes, starting concentrations of HEV, cell types, HEV detection methods, and so on. Starting/spiking concentrations of HEV could affect inactivation kinetics. The cell culture associated with HEV has difficulties, such as the limited susceptibility of cell lines and the potential for adaptation [53]. Schielke et al. [54] reported the incomplete inactivation of HEV even at 95 °C, which contrasts with other studies. They used RNase treatment followed by quantitative real-time RT-PCR to differentiate disassembled from intact viral particles. The presence of intact viral particles does not necessarily equate to infectivity. It is possible that studies relying on real-time PCR may also assess impaired viral particles. It is possible that the viability procedure could explain some discrepancies [60].

The data of the present article may help to estimate the stability of HEV in the environment or food. It is shown here that heat treatment at 95 °C for 10 min prior to the inoculation of PLC/PRF/5 cells could lead to HEV RNA being undetectable throughout the observation period of 50 days after inoculation [49]. At present, heat inactivation at 95 °C for 10 min is the safest approach for the inactivation of HEV (Figure 1a) [11]. Further studies will be needed to confirm this observation.

We reviewed the thermal inactivation of four major HEV genotypes: HEV-1, HEV-2, HEV-3, and HEV-4. It has been reported that HEV-7 is also a causative agent for human hepatitis E [68,69]. The consumption of camel-derived food products may link to post-transplantation chronic hepatitis E [68]. Further studies of the heat inactivation of HEV-7 are needed.

There are two forms of HEV particles: eHEV and neHEV [70]. As cell-culture-derived HEV is often eHEV, it may be possible that eHEV has a similar thermal inactivation pattern as neHEV, which mainly exists in bile or feces. Further studies are needed regarding this point.

The consumption of innards, offal, organ meat, and various other meats is linked to hepatitis E [71], and eating them represents a risk factor for HEV infection [72]. The heat inactivation of HEV is also important before eating the innards, offal, organ meat, and a variety of other meats (aside from liver) from pigs.

At present, HEV transmission through the food chain is a critical source of infection; HEV-3 and HEV-4 have wide host ranges and pose a great risk of infection, especially for high-risk groups, and surveillance is important to examine the prevalence and infectivity of HEV-3 and HEV-4 in food products [13]. The bloodborne transmission of HEV poses a great risk, especially for high-risk groups [13], and HEV screening in blood donors is also important [19,35,36]. HEV countermeasures, including vaccines and antivirals, should be developed [73].

## 6. Future Perspectives

Between the harvesting and consuming stages, in general, food can become contaminated by bacteria, parasites, and viruses, including HEV [74]. To identify the relevant foodborne pathogens, there are several conventional and advanced methods. Culture-, biochemical test-, immunological-, and nucleic acid-based methods represent the conventional ones, while hybridization-, array-, spectroscopy-, and biosensor-based methods are more advanced (Table 2) [74,75]. The gold standard detection method for foodborne pathogens in the food industry is based on rapid PCR screening [76].

Bennett et al. [50] reported that when applying the recommended cooking times (71 °C for 2 min; 71 °C for 30 sec; and no cooking), the per serving exposure outcomes for HEV in pig liver (97.5th percentile (95% CI)) were expected to be 109 copies/g, 376 copies/g, and 1157 copies/g, respectively, meaning that heating at 71 °C for short time does not completely achieve inactivation. Heating at 70–72 °C for 2 min significantly reduces HEV infectious titers but often does not result in a >4 log_10_ decrease [75].

Detailed analyses of HEV have been hampered by the absence of viral cell culture systems capable of detecting low-titer viruses. It is possible that foodborne HEV may not replicate efficiently in conventional in vitro culture systems. At present, the thermal inactivation of HEV-contaminated food products requires heating at temperatures above 70 °C for at least 23 min to achieve the effective inactivation of HEV [58]. However, the thermal stability of HEV varies significantly depending on the viral strain, food matrix, and specific heating condition [75]. Heating at 95 °C for 10 min may represent a more reliable method for ensuring the complete inactivation of HEV in food. Therefore, it is critical that consumers are informed about appropriate heat-treatment protocols and durations to effectively eliminate HEV from food products.

## 7. Conclusions

A vaccine for hepatitis E is currently available in China and Pakistan. The effective inactivation of infectious HEV is critical for ensuring the safety of drinking water and food products. HEV RNA has been detected in various porcine-derived food products, including intestines and livers, suggesting the potential for these products to act as sources of HEV transmission. The thermal stability of HEV has been demonstrated in both cell culture systems and pig bioassays. The heat treatment of HEV-contaminated foods at 95 °C for 10 min or 70 °C for at least 23 min has been suggested as an effective strategy for viral inactivation. However, standardized thermal inactivation methods for HEV in plasma-derived products have not yet been established. NAT is useful for preventing transfusion-transmitted HEV infection.

## Figures and Tables

**Table 1:** The detection of hepatitis E virus RNA in pig products, including intestines and livers, which are sold in grocery stores.

Countries	Foodstuffs	Prevalence (%)/Total N	Year, Refs.
Japan	Raw pig liver	7 (1.9%)/363	2003, [38]
Japan	Raw pig liver	12 (4.9%)/243	2014, [40]
United States	Pig liver	14 (11%)/127	2007, [45]
Netherlands	Pig liver	4 (6.5%)62	2007, [47]
India	Pig liver	2 (0.8%)/240	2008, [48]
Germany	Pig liver	8 (4%)/200	2011, [41]
Germany	Pig products (liver, liver sausages, liver pate samples, etc.)	13 (10%)/130	2021, [42]
France	Raw figatellu (a traditional pig liver sausage)	7 (58.3%)/12	2010, [49]
Italy	Raw pig liver sausages	10 (22.2%)/45	2015, [43]
Italy	Dry pig liver sausages	1 (4.3%)/23	2015, [43]
Colombia	Pig liver from slaughterhouses	62 (41.3%)/150	2015, [44]
Columbia	Pig liver from grocery stores	25 (25%)/100	2015, [44]
Ireland	Pig products	9 (4.8%)/188	2024, [50]
Ireland	Pig liver	6 (24%)/25	2024, [50]
Ireland	Fermented sausage (pig)	1 (2.0%)/49	2024, [50]
Ireland	Pig sausage (27 g)	1 (1.5%)/65	2024, [50]
Ireland	Pig sausage (72 g)	1 (2.0%)/49	2024, [50]

N, number. Refs., references.

## Data Availability

Not applicable.

## Abbreviations

The following abbreviations are used in this manuscript:

HEV	hepatitis E virus
NAT	Nucleic acid testing
CPE	cytopathic effect
ffu	focus-forming units
GEs	genome equivalents
RT-PCR	Reverse transcription–polymerase chain reaction
TCID_50_	A 50% tissue culture infective dose
